# Inhibition of protein kinase C promotes dengue virus replication

**DOI:** 10.1186/s12985-016-0494-6

**Published:** 2016-03-01

**Authors:** Warobon Noppakunmongkolchai, Teera Poyomtip, Thichakorn Jittawuttipoka, Natthanej Luplertlop, Anavaj Sakuntabhai, Sarin Chimnaronk, Siwanon Jirawatnotai, Rutaiwan Tohtong

**Affiliations:** Department of Biochemistry, Faculty of Sciences, Mahidol University, Bangkok, Thailand; Department of Microbiology, Faculty of Sciences, Mahidol University, Bangkok, Thailand; Department of Microbiology and Immunology, Faculty of Tropical Medicine, Thailand Functional Genetics of Infectious Diseases Unit, Institute, Mahidol University, Bangkok, Thailand; Institute Pasteur, Functional Genetics of Infectious Diseases Unit, Paris, France; Centre National de la Recherche Scientifique (CNRS), URA3012, F-75015 Paris, France; Institute of Molecular Biosciences, Mahidol University, Salaya, Nakhon Pathom Thailand; Systems Biology of Diseases Research Unit, Faculty of Science, Mahidol University, Bangkok, Thailand; Laboratory for Systems Pharmacology, Department of Pharmacology, Faculty of Medicine Siriraj Hospital, Bangkok, Thailand

**Keywords:** Dengue virus (DENV), Protein kinase C (PKC), Phosphorylation, Non-structural protein 5 (NS5), Viral replication, Flavivirus

## Abstract

**Background:**

Dengue virus (DENV) is a member of the *Flaviviridae* family, transmitted to human *via* mosquito. DENV infection is common in tropical areas and occasionally causes life-threatening symptoms. DENV contains a relatively short positive-stranded RNA genome, which encodes ten viral proteins. Thus, the viral life cycle is necessarily rely on or regulated by host factors.

**Methods:**

*In silico* analyses in conjunction with *in vitro* kinase assay were used to study kinases that potentially phosphorylate DENV NS5. Potential kinase was inhibited or activated by a specific inhibitor (or siRNA), or an activator. Results of the inhibition and activation on viral entry/replication and host cell survival were examined.

**Results:**

Our *in silico* analyses indicated that the non-structural protein 5 (NS5), especially the RNA-dependent RNA polymerase (RdRp) domain, contains conserved phosphorylation sites for protein kinase C (PKC). Phosphorylation of NS5 RdRp was further verified by PKC *in vitro* kinase assay. Inhibitions of PKC by a PKC-specific chemical inhibitor or siRNA suppressed NS5 phosphorylation *in vivo,* increased viral replication and reduced viability of the DENV-infected cells. In contrary, activation of PKC effectively suppressed intracellular viral number.

**Conclusions:**

These results indicated that PKC may act as a restricting mechanism that modulates the DENV replication and represses the viral outburst in the host cells.

**Electronic supplementary material:**

The online version of this article (doi:10.1186/s12985-016-0494-6) contains supplementary material, which is available to authorized users.

## Background

DENV infection is among major life-threatening infectious diseases in tropical countries. A fraction of infected human will develop severe life-threatening symptoms, which are recognized as dengue hemorrhagic fever (DHF). This composes of bleeding, low levels of blood platelets and blood plasma leakage. It can develop into dengue shock syndrome (DSS), where dangerously low blood pressure occurs. These are normally followed by mortality, since there is no specific management for it, except the symptomatic treatments. Currently, there is no effective vaccine that can prevent the infection. Therefore, information on the pathogenesis and the biology of the virus is required to build novel strategies to reduce the death rate caused by this virus.

DENV is a relatively simple virus. Its genome encodes 10 viral proteins, including 3 structural proteins; envelope (E), capsid, prM, and 7 non-structural (NS) proteins; NS1, NS2A, NS2B, NS3, NS4A, NS4B, and NS5. To reproduce in the host cells, DENV requires host’s components to propagate [1–4]. Thus, virus-host interaction is one of the major processes that influence viral life cycle, and is being extensively investigated.

Accumulating evidences have shown that many viral proteins interact with host proteins. Viral proteins, such as NS1, NS4A, NS5, E, and capsid, were demonstrated by yeast two-hybrid or by co-immunoprecipitation coupled with Mass spectrometry to physically associate with host proteins [5–7]. Many of these interactions were proven to be physiologically relevant [6, 8, 9]. Functional interactions, such as phosphorylation and methylation, are also subjected for examinations. Recent evidences indicated that host proteins functionally interact with, and regulate the viral proteins, and *vice versa* [10–13].

NS5s from various flaviviruses have long been distinctively characterized as a phosphoprotein which contains several possible phosphorylation sites [14], suggesting a significance of this phospho regulation for the viruses. The protein is composed of 2 functional domains, namely the N-terminal methyltransferase, which catalyzes guanine N-7 and ribose 2’-OH methylations of the 5’ terminal cap 1 structure (m7GpppAmG) during viral cap formation [15], and the C-terminal RNA-dependent RNA polymerase (RdRp), which is an enzyme that synthesizes viral RNAs during viral replication [16].

Identification of kinases that responsible for the phosphorylation has been a topic of interest. Recent reports showed that NS5 is phosphorylated by Casein Kinase I (CK I) or protein kinase G (PKG) at the methyltransferase domain [10, 17]. The phosphorylations appeared to be essential for the normal function of NS5, since blocking the phosphorylation either by chemical inhibitor or amino acid substitution of the potential phosphorylation site suppressed viral replication and viral production [17]. These findings also suggested that NS5 is a central protein, which mediates functional interactions between host and virus proteins. Detailed study of NS5 phosphorylation may provide a better understanding of the viral life cycle, and may lead to intervention approaches that are based on viral-host interaction. In this study, we performed *in silico* analyses to identify human kinases that potentially regulate DENV2 NS5. We identified that there were several possible PKC phosphorylation sites on the NS5. We then also studied the physiological relevance of the phosphorylations for the virus and host cells.

## Results

### NS5 contains possible protein kinase C phosphorylation sites

**Fig. 1:**
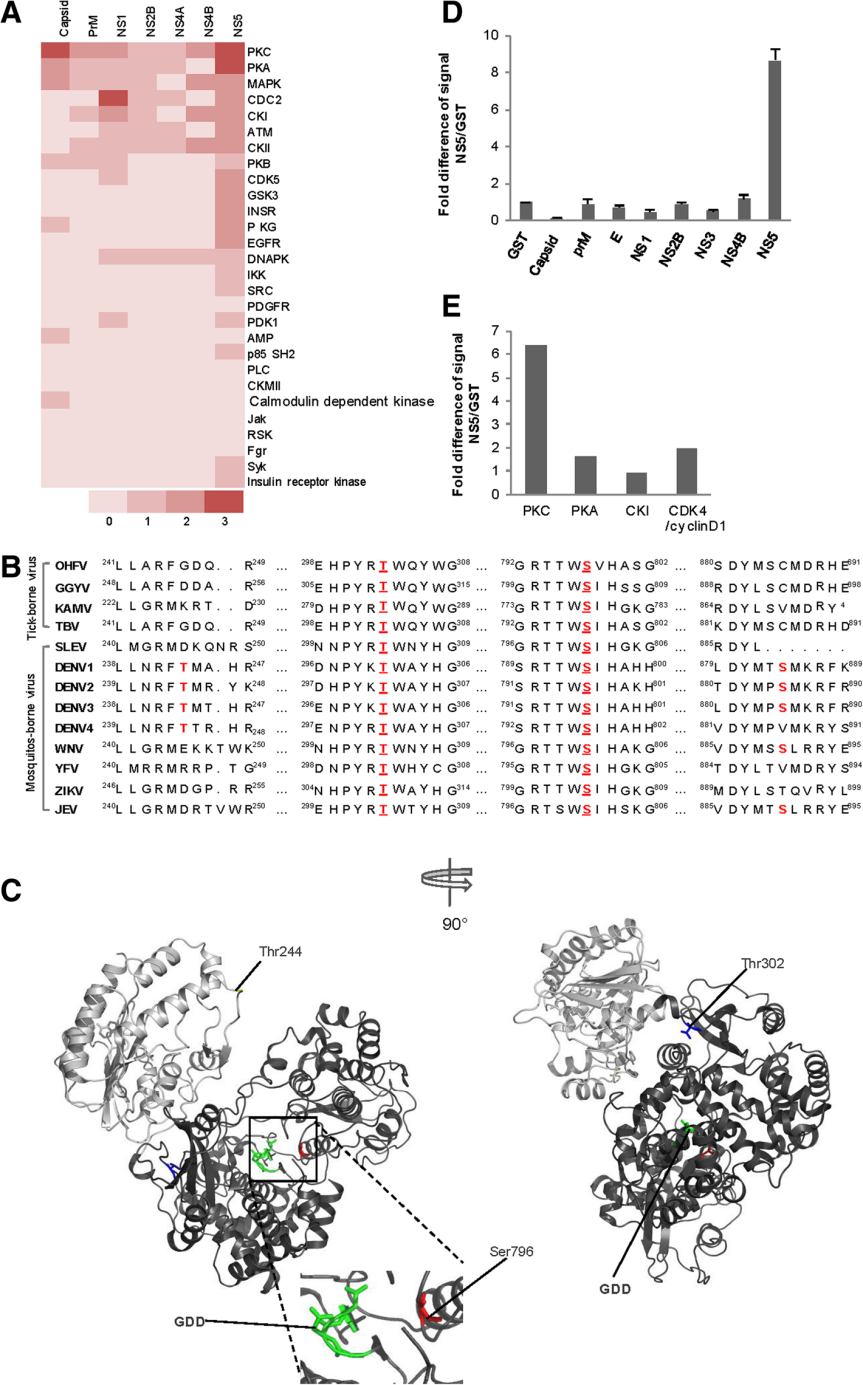
*In silico* analyses revealed possible protein kinase C phosphorylation sites on dengue NS5. **a** Prediction of possible NS5 kinases. A heat map showed possible human kinases that phosphorylate DENV2 NS5 based on *in silico* predictions by Scansite™, NetPhosK 2.0, and KinasePhos 2.0 (upper panel). Names of possible kinases were listed vertically on the right of the panel. DENV2 proteins were listed horizontally on the top. Color codes (lower panel) indicated numbers of hit from the predicting algorithms; from 0 (very light red) to 3 (dark red). **b** Sequence conservation of the predicted PKC phosphorylation sites across flaviviruses. Partial amino acid sequences of flaviviruses were listed as indicated by name; Omsk hemorrhagic fever virus (OHFV), Gadgets Gully virus (GGYV), Kama virus (KAMV), and tick-borne encephalitis virus (TBV), St. Louis encephalitis virus (SLEV), dengue 1–4 (DENV1–4), West Nile virus (WNV), yellow fever virus (YFV), Zika virus (ZIKA), Japanese Encephalitis virus (JEV). Predicted PKC phosphorylation sites were showed in RED. In addition, Thr302, and Ser796 that were conserved were underlined. **c** Three-dimension models of DENV2 NS5 protein. Colored in light gray is the methyltransferase domain, dark gray is the RNA-dependent RNA polymerase (RdRp) domain. Highlighted in yellow, red, and blue are possible PKC phosphorylation sites; Thr244, Ser796, andThr302, respectively. Highlighted in fluorescent green is the GDD RNA polymerase active site. **d** PKC *in vitro* kinase assays. Purified DENV2 proteins were used as substrate. Purified GST was used as a non-relevant control for the reaction. Bars represented means of 3 independent experiments, error bars; S.D. **e** *In vitro* kinase assays, several human kinases were tested including PKC, PKA, CKI, and CDK4/cyclin D1, as indicated. The signal was normalized by the signal from reactions with GST. Bars represented data from a representative experiment

To investigate possible amino acids on NS5 that might be phosphorylated by human kinases, we analyzed amino acid sequence of dengue serotype 2 (DENV2) NS5 by three phospho-algorithms; Scansite™ [18], NetPhosK 2.0 [19], and KinasePhos 2.0 [20]. In accordance with the previous notion describing NS5 as phosphoprotein, the analyses revealed many potential candidate phosphorylation sites on NS5 for several kinases, when compared to other DENV proteins (Fig. 1a). Protein kinases predicted to phosphorylate NS5 by at least one algorithm included PKC, PKA, CDKs (CDCs), MAPK, ATM, CK II, PKB, and others (Fig. 1a). Our analyses also identified 2 kinases that were previously reported to phosphorylate NS5, i.e. Casein Kinase I (CK I), and protein kinase G (PKG) [10, 17, 21]. Among the candidates, we focused on possible functional interaction between NS5 and PKC (predicted by all 3 algorithms). We found that 4 potential phosphorylation sites, in particular Thr244, Thr302, Ser796, and Ser885, were recognized by at least 2 predicting algorithms on DENV2 NS5. Among the 4 candidates, Thr302 and Ser796 are conserved in all vector-borne flaviviruses (Fig. 1b). Thr244, and Ser885 were not in the other mosquito-borne or tick-borne viruses, but were rather limited within the DENVs (Fig. 1b). While Thr244 resides in the methyltransferase domain of NS5, Thr302 Ser796 and Ser885 reside in the RdRp domain of NS5. These 4 candidate sites are exposed to solvent, thus, are potentially be phosphorylated (Fig. 1b, c).

Since RdRp domain contains several of the possible phosphorylation sites for PKC, we set up a PKC *in vitro* kinase assay using purified recombinant DENV2 RdRp domain, containing Thr302 Ser796 and Ser885, as a substrate. The kinase assay showed that PKC efficiently phosphorylated RdRp *in vitro*. The signal was approximately 9 folds higher than that of GST (used as non-relevant control). Under these conditions, PKC kinase assay gave a very low signals for the other DENV proteins, such as capsid, prM, E, NS1, NS2B, NS3, NS4B (Fig. 1d). Interestingly, protein kinase C appeared to produce relatively high phosphorylation signal compared with the other potential NS5 kinases, namely PKA, CK I, and CDK4/cyclinD1 (Fig. 1e). Thus, PKC can efficiently phosphorylated the NS5 RdRp domain *in vitro*. Of note, although the methyltransferase domain contains only one possible PKC phosphorylation site, Thr244, which is not conserved in all of the vector-borne flaviviruses, it is entirely possible that this site may be phosphorylated by PKC. We have yet to test this possibility.

### Silencing of PKCα reduced Ser and Thr phosphorylation of NS5 in vivo

**Fig. 2:**
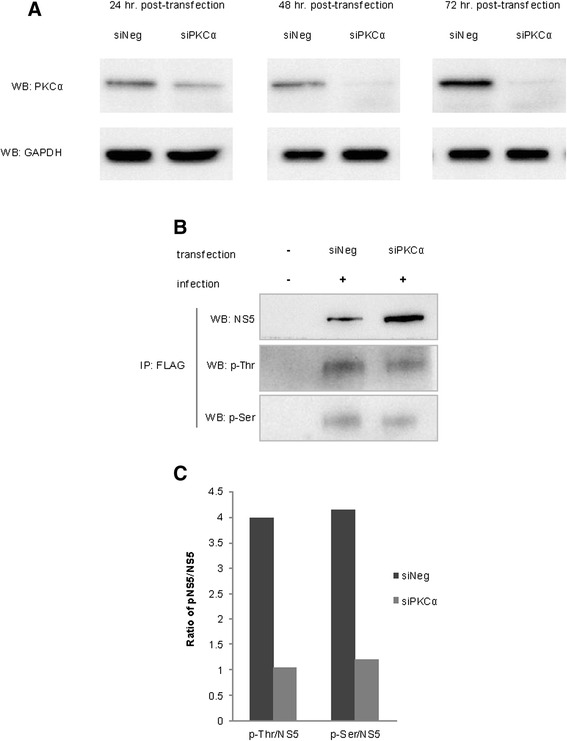
Inhibitions of PKC by PKCα siRNA or a chemical inhibitor (BisI) suppressed NS5 RdRp phosphorylations. **a** PKCα-specific siRNA depleted endogenous PKCα at 24, 48, 72 h after siRNA transfection. A pool of PKCα siRNAs (siPKCα) was transfected into the BHK-21 cells. The levels of PKCα were detected by PKCα immunoblotting. A non-targeting siRNA (siNeg) was used as a control. GAPDH immunoblots were used as a loading control. **b** PKCα depletion by siRNA downregulated NS5 Threonine and Serine phosphorylations. BHK-21 cells were infected with recombinant DENV expressing His-FLAG-tagged NS5. The His-FLAG-tagged NS5 was pulled down (IP) from BHK-21 cell lysate by anti-FLAG antibody, and Threonine/Serine phosphorylations (p-Thr/p-Ser) were examined by p-Thr or p-Ser-specific antibodies. **c** Quantifications of p-Thr/NS5 and p-Ser/NS5 from (**b**)

To investigate the functional interaction between host PKC and DENV NS5 *in vivo*, we depleted PKCα using PKCα-specific siRNAs and examined the level of NS5 phosphorylation *in vivo*. We first, tested the efficiency of siRNA-mediated PKC depletion. We found that at 24, 48, and 72 h after PKC-specific siRNA transfection, more than 70 % of the endogenous PKC levels were downregulated (Fig. 2a), while host cell viability was unaffected (data not shown). We selected to examine NS5 phosphorylation at the 24 h after siRNA transfection. We expressed His-FLAG-tagged NS5 in host cells by infecting the cells with recombinant DENV expressing the His-FLAG-NS5 protein (etDENV), then pulled down the NS5 using anti-FLAG antibody. The phosphorylation on the His-FLAG-NS5 was analyzed by anti phospho-Ser and phospho-Thr immunoblottings. We found that under this condition, silencing of the PKC clearly decreased Serine and Threonine phosphorylations of NS5 (Fig. 2b, c).

### Inhibition of PKC by BisI reduced Ser and Thr phosphorylation of NS5 in vivo

**Fig. 3:**
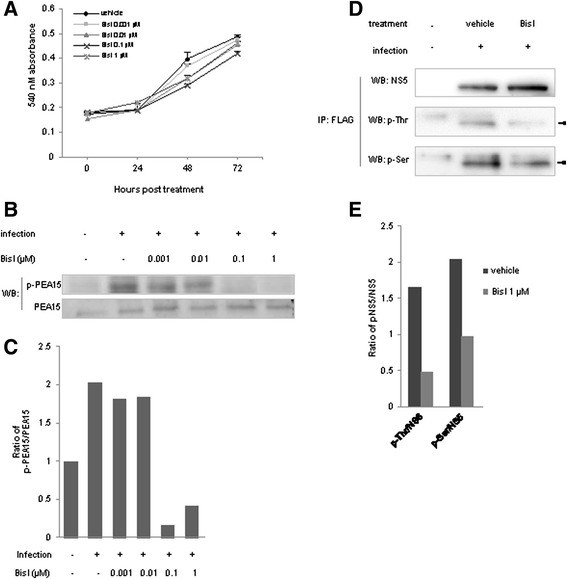
Inhibition of PKC by BisI. **a** Inhibition of PKC by BisI did not alter host cell growth. HepG2 cells were treated with various concentrations of BisI. Average cell viability from right after the treatment (0 h), 24, 48, and 72 h was monitored by MTT assays. Error bars represented S.D. from 3 independent experiments. **b** BisI effectively inhibited PKC activity. Phosphorylation of PEA15, a known substrate of PKC. Levels of phospho-PEA15 at 24 h after BisI treatment were examined by immunoblotting using a phospho-PEA15 (p-PEA15) specific antibody. Concentrations of BisI used were indicated. PEA15 immunoblot was used to examine the total amount of PEA15. **c** Quantification of p-PEA15 from **b** Amounts of p-PEA15 were normalized by total PEA15. **d** PKC inhibition by 1 μM of BisI downregulated NS5 Threonine and Serine phosphorylations. BHK-21 cells were infected with recombinant DENV expressing His-FLAG-NS5, and tagged protein was then pulled down by anti-FLAG antibody. The p-Thr and p-Ser were examined by immunobloting as in Fig. 2b. Arrow heads indicated specific p-Thr or p-Ser signals. **e** Quantifications of p-Thr/NS5 and p-Ser/NS5 from (**d**)

To confirm the effect of PKC depletion on NS5 phosphorylation, we inhibited PKC activity of host cells using bisindolylmaleimide I (BisI), a specific chemical inhibitor of conventional and novel PKCs. We found that various concentrations of BisI, (up to 1 μM of BisI) were not toxic to the cells for at least up to 72 h (Fig. 3a). As early as 24 h after treatment, 0.1 and 1 μM of BisI clearly suppressed the activity of PKC, as indicated by a complete loss of the phosphorylation of PEA15, an established PKC substrate (Fig. 3b, c). Importantly, FLAG immunoprecipitation of the tagged-NS5 followed by phospho-Ser or phospho-Thr immunoblotings showed that Serine and Threonine phosphorylations were decreased, when the cells were treated with non-toxic dose (1 μM) of BisI (Fig. 3d, e).

### PKC blockage enhanced DENV viral replication, but not viral entry into host cells

**Fig. 4:**
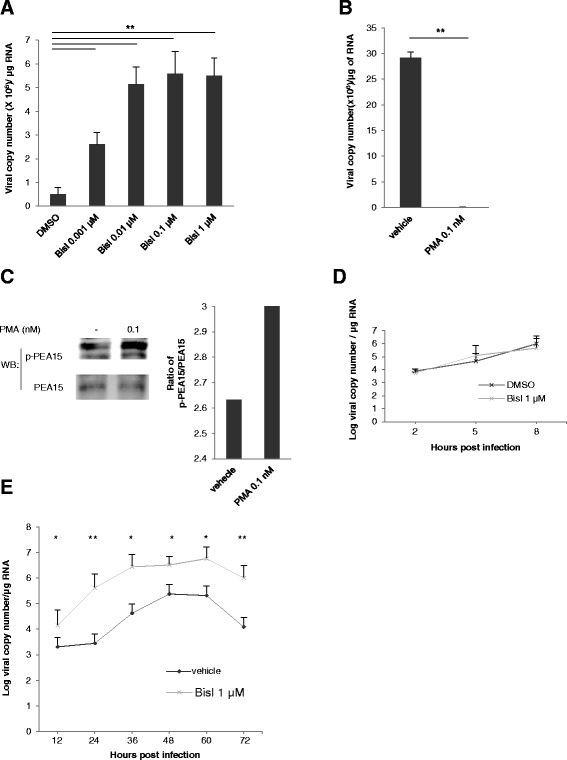
PKC activity restricted DENV viral number in host cells. **a** Viral copy numbers in HepG2 cells treated with various BisI concentrations. Cells were treated with various concentrations of BisI for 30 min, prior to a 1.5-h infection by DENV strain 16681. Cells were kept in medium with BisI for a total of 24 h, before harvesting. Viral copy numbers were quantified by quantitative PCR. Bars represent means viral copy number from 3 independent experiments. Error bars represent S.D., **; *p-value* ≤ 0.01. Concentrations of BisI used were not toxic to the host cells. **b** Reduction of DENV copy number in host cells by PKC activator. HepG2 cells were treated with 0.1 nM of a PKC activator PMA or vehicle for 30 min, prior to a 2-h infection by DENV strain 16681 (MOI of 5). Cells were then kept in the activator (or vehicle) for 24 h before harvesting to quantify the viral copy numbers. Bars represent means of 3 independent experiments. Error bars represent S.D., **; *p-value* ≤ 0.01. **c** PMA promoted PKC activity. Levels of total PEA15 and phospho-PEA15 at 24 h after PMA treatment were examined using immunoblotting (left panel). Ratios of the signals p-PEA15/PEA15 from the immunoblots were shown on the right. **d** BisI had no effect on entering of virus in to the host cells (HepG2). Average viral copy numbers at the early time points after viral infection, 2, 5, 8 h. Viral infection and BisI treatment was performed as indicated in **a** Three independent experiments were performed. Error bars represent S.D. **e** DENV copy numbers in HepG2 cells during indicated time pointed were represented as mean of 3 independent experiments. Error bars represent S.D., *; *p-value ≤ 0.05*, **; *p-value* ≤ 0.01. BisI treatment and viral infection was performed as indicated in (**a**)

To examine the biological effects of PKC blockage on the viral replication, we quantitated the viral copy number in the host cells pre-treated with BisI using RT-qPCR. BisI, which blocked the PKC activities, significantly increased the viral copy of DENV2 strain 16681 in human hepatocellular carcinoma HepG2 cells, with the highest copy number of the virus attained by the 0.1 and 1 μM BisI treatments (Fig. 4a). On the contrary, we detected a very lowering of dengue virus copy number in HepG2 cells pretreated with 0.1 nM of a PKC activator Phorbol 12-myristate 13-acetate (PMA) (Fig. 4b). We found that, at this dose of PMA, activity of PCK was induced indicated by an upregulation of phospho-PEA15 (Fig. 4c).

To further investigate whether the PKC inhibitor altered DENV entry into the host cells, we assessed the viral copy number in the cells at early time points, i.e. at 2, 5 and 8 h after viral infection. During these time points, the viral replication was not yet fully operated. Therefore, the viral copy number would represent the number of virus entering the host cells after the infections [22]. We found that the amounts of virus in BisI-treated cells were comparable to those in the vehicle-treated cells, during these early time points (Fig. 4d). Therefore, it is likely that the activity of PKC was not involved with DENV entry into the host cells, and inhibition of PKC activity does not interfere with this process.

In contrast to the results from early time points, viral copy numbers at the later time points were augmented by at least 10 folds (after 12 h of BisI-treatment), when compared to that of the vehicle-treated cells. The viral copy numbers continued to be higher at every time point examined until 72 h post infection (Fig. 4e).

These results indicated that inhibition of the PKC activity promotes viral replication, showing in an increase of the viral copy number, whereas stimulation of the PKC activity may be involved in suppression of the DENV replication in the host cells.

**Fig. 5:**
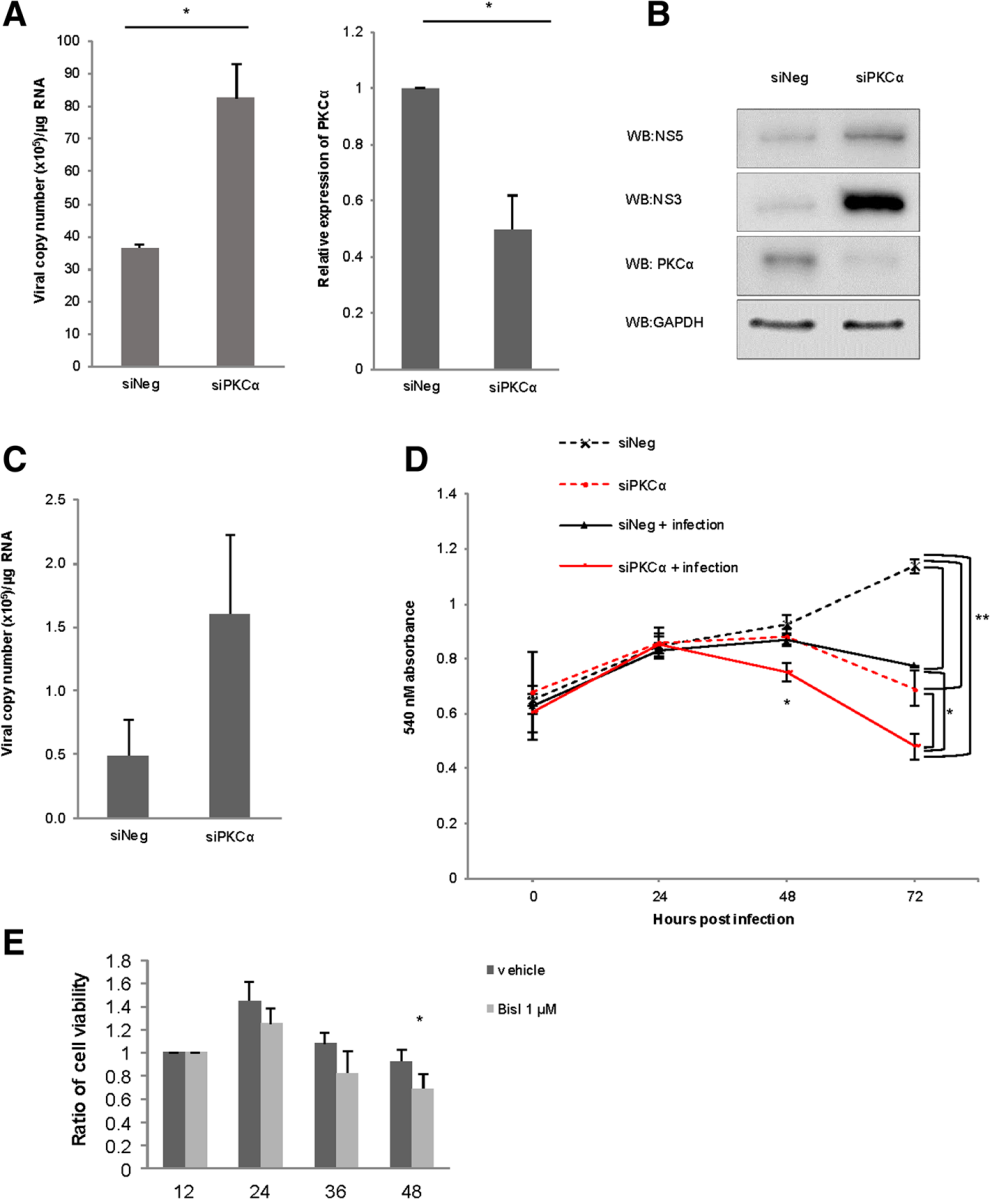
Depletion of PKC increased viral-induced host cell damage. **a** siRNA-mediated depletion of PKCα resulted in increased intracellular DENV copy number. HepG2 cells were transfected with PKCα-specific siRNA (siPKCα) or non-targeting siRNA (siNeg) for 24 h prior to a 2-h infection of DENV strain 16681. After 24 h, cells were harvested. PKCα mRNA and DENV viral copy numbers were quantified by quantitative PCR. Left; bars represent viral copy numbers in the host cells treated with siRNA indicated. Right; bars represent means expressions of PKCα mRNA from 3 independent experiments. Error bars represent S.D., *; *p-value* ≤ 0.05. **b** Expressions of PKCα, DENV NS3, and NS5 were examined by immunoblots using indicated antibodies. GAPDH immunoblot was used as loading control. **c** Average DENV viral copy numbers in culture mediums from PKCα-depleted HepG2 cells (siPKCα), compared to control cells (siNeg). Bars represent means of 3 independent experiments. Error bars represent S.D. The viral copy numbers were determined by RT-qPCR. **d** Depletion of PKCα decreased the viability of DENV-infected cells. HepG2 cells were treated as indicated. The 24 h post transfection, cells were infected with DENV. Cell viabilities were examined using MTT assay. Graphs showed average of 3 independent experiments. Error bars represent S.D. *; *p-value* ≤ 0.05, **; *p-value* ≤ 0.01. **e** Inhibition of PKC by BisI decreased viability of DENV-infected cells. HepG2 cells were treated with 1 μM BisI for 30 min, prior to a 2-h infection by DENV strain 16681. Cell viabilities were determined by tryptan blue staining at indicated time points. Bars represent means of 3 independent experiments. Error bars represent S.D., *; *p-value* ≤ 0.05

Promotion of the viral replication by PKC blockage was also confirmed using siRNA-mediated silencing of PKCα. This also resulted in a significant increase of dengue viral copy number and the amounts of viral NS5 and NS3 proteins in host cells (Fig. 5a, b). Although not statistically significant, we detected an increase of secreted virus into the culture medium of BisI-treated cells as well (Fig. 5c).

### Inhibition of PKC reduced host cell viability

The above data showed that depletion of PKC significantly enhanced the dengue viral replication in the host cells. To further assess whether PKC depletion also had an effect on the host cells, we monitored growth of the infected HepG2 cells in the presence or absence of PKC depletion. To this end, HepG2 cells, which either pretreated with non-targeting (control) siRNA or PKC-specific siRNA, were infected with DENV2 16681. We found that, viability of the non-targeting siRNA-treated control cells without viral infection consistently increased over 3 days (Fig. 5d, dashed black line). Infection of dengue virus, or PKC siRNA transfection alone, significantly reduced HepG2 cell viability by 72 h after the PKC knockdown (Fig. 5d, dashed red line and solid black line, respectively). Importantly, depletion of PKC by siRNA together with DENV-infection accelerated suppression of HepG2 cell viability to 48 h after transfection. The loss of host cells became more clearly evident at the 72 h time point (Fig. 5d, solid red line). Inhibition of PKC by BisI also caused a marked reduction of host cell viability at 48 h after DENV infection (Fig. 5e). Altogether, these data indicated that inhibition of PKC led to an enhancement of the DENV growth and suppression of host cell viability.

## Discussion

Significance of the interaction between virus and host cells have been established, especially that of positive-strand RNA viruses, such as the DENV and the host cells. The simplicity of the viruses, and limited number of viral proteins have made the host factors indispensable for the virus. Physical interactions between host proteins and several viral proteins have been widely studied. Yeast-two-hybrid and immunoaffinity-purification/Mass spectrometry experiments revealed extensive physical networks of viral-host interactomes [23]. Biochemical reactions between biomolecule are often transient, and sometimes fastidiously hard to detect. Hence, there are fewer numbers of report describing functional interactions among the host-viral proteins. However, functional interactions are of importance, since all of them are virtually regulative and physiologically meaningful for the co-existing of the virus and the host cell.

In this study, we used an *in silico* screening approach, which relied on three different phospho-scanning tools, to search for novel phosphor regulatory sites on DENV NS5 protein. None of the screening tools to these days can accurately predict phosphorylation for all kinases. This is because many kinases do not have a clear consensus site. Thus, we incorporated results from all of the tools, hoping to identify the most possible phospho-sites on the DENV proteins. From our *in silico* analyses, we found that despites NS5, capsid was also predicted as a potential substrate for PKC (Fig. 1a). However, in a subsequent experiment, capsid was shown to be a poor PKC substrate in the *in vitro* kinase assay. This implicated that a verification of the *in silico* prediction is indispensable.

Flaviviral NS5 was shown to be a phosphoprotein and is regulated by human kinases [11, 17]. Phosphorylation by host factors in many cases are required for normal function of NS5 proteins, for example phosphorylation of NS5 was shown to involve with NS3-NS5 complex formation [12]. PKG-NS5 interaction is required for viral replication [17, 21]. This interaction was shown to be involved with the NS5 methyltransferase domain. CK I was also shown to phosphorylate the same domain on NS5 and regulate methyltransferase function, hence the production of virus in the host cells [10].

On the other hand, some NS5 phosphorylations have been shown to inhibit the function of NS5. Previous report has shown that the NS5 methyltransferase function of Yellow fever virus (YFV) was suppressed by phosphorylation [24].

In addition, the RdRp domain on NS5 was predicted to be phosphorylated by CK II on residues distinct from those phosphorylated by PKC. The CK II phosphorylation site was located in the 37-amino-acid linker interdomain of NS5 (residues 369 to 405), which contains the nuclear localization signal (NLS). Phosphorylation at this site has been shown to inhibit of NS5 nuclear targeting, resulting in retention of the RdRp in the cytoplasm [11].

Here, we showed that phosphorylation in the NS5 by PKC is inhibitory and is physiologically relevant. Inhibition of this phosphorylation reaction resulted in a significant increase of viral number and reduction of host cell viability. Conversely, activation of PKC by PMA suppressed intracellular DENV production. Thus, it is possible that PKC might represent an intracellular restricting mechanism that prevents viral outburst in the host cells. In accordance with our result, a recent report showed that a siRNA screening identified *PIKC1*, an protein kinase Cα-interacting protein as a host factor that restricting DENV virus [25].

**Fig. 6:**
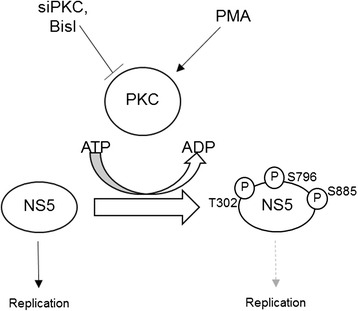
Inhibition of NS5 phosphorylation by PKC promotes viral replication. PKC is a host factor, which restricts intracellular DENV2 number by phosphorylating viral NS5. Inhibition of PKC by PKC inhibitor (BisI) or PKC-specific siRNA (siPKC) results in increased viral copy number

In this study, we have shown by *in vitro* kinase assay that the RdRp domain within NS5 was a substrate for PKC. This domain contains three PCK phosphorylation sites (Thr302, Ser796, and Ser885). We hypothesized that some of the PKC phosphorylation sites might be regulatory site for RdRp’s function. For example, Ser796, is located near the active site of the RNA polymerase (GDD site) (Fig. 1c) [26]. Thus, hyperphosphorylation of these sites by PCK may interfere with the RNA polymerase function of NS5 RdRp, leading to suppression of DENV replication (Fig. 6). However, further experiments are required to validate the model. Of note, PKC has been shown to be involved with cellular apoptosis. In most contexts, such as in melanoma cell lines [27], bladder carcinoma cell lines [28], glioma cells [29], and salivary gland epithelial cells [30], PKC especially PKCα appeared to inhibit apoptosis (except, in the LNCaP prostrate cells in which expression of PKCα promotes apoptosis) [31]. Loss of PKCα activity either induces death, or sensitizes cells to death signals. Although, our experiments in hepatocellular carcinoma cells found that the inhibitions of PKC activity did not cause any notable cell death under the conditions, it is possible that PKC may also contribute to suppress host cell loss by specifically inhibiting apoptosis signal triggered by DENV infection.

## Conclusions

PKC may act as a restricting mechanism that modulates the DENV replication and represses the viral outburst in the host cells.

## Methods

### In vitro kinase assay

The His-tagged NS5 RdRp protein was purified from bacteria expression following Kamkaew et al. [32]. Dengue Capsid, prM, E, NS1, NS2B, NS3 and NS4B coding DNA were amplified by PCR from pD2-IC plasmid containing DENV2 genome [33]. The PCR products were inserted into pGEX-5X-3 plasmid (GE Healthcare Bio-Sciences). *E.coli* BL21 (DE3) was used to express dengue proteins according to standard protocol recommended by the manufacturer. The assay use 1 μg DENV proteins as substrate in a reaction with 75 ng of purified human PKC (Sigma). Cdk4/cyclinD1, PKA, CK I kinases were purchased from Calbiochem, Sigma and Promega, respectively. The kinase buffers and the reactions were prepared and performed as recommended by the manufacturer’s protocols. After the kinase reactions, the substrates were run on a 12 % SDS-PAGE gel. Expected bands of substrates were visualized by Coomassie blue staining and were excised from the gel to measure the ^γ-32^P incorporation. The ^γ-32^P was detected by scintillation counter (Perkin Elmer).

### Cell lines and culture conditions

The human liver hepatocellular carcinoma cell line HepG2 was a kind gift from Dr. Limjindaporn T, Faculty of Medicine, Siriraj Hospital, Mahidol University. The Baby hamster kidney cell line (BHK-21) was a kind gift from Dr. Leardkamolkarn V, Faculty of Sciences, Mahidol University. They were cultured in Dulbecco’s Modified Eagle Medium (DMEM) supplemented with 10 % heat-inactivated fetal bovine serum (FBS), 100 U/ml penicillin G and 100 μg/ml streptomycin (Invitrogen). Cells were grown at 37 °C with 5 % CO_2_ in a humidified incubator.

### Recombinant epitope-tagged DENV2

Construction of the recombinant DENV harboring the His-FLAG-tagged NS5 DENV (etDENV) was described previously [34]. Briefly, the cDNA from DENV serotype 2 strain 16681 in the pD2-IC plasmid [33] was used as the template for the insertion of the epitope tag. The TAP tag comprised of an octahistidine (His8) tag, a tri-glycine spacer, and FLAG sequence (DYKDDDDK) was inserted into the NS5 gene in pD2-IC via a standard QuikChange procedure. The positive clone containing the tag was verified by DNA sequencing. Wild-type and tagged DENV RNAs were synthesized according to a previous report [33]. Assay for viral kinetics was performed as follows: BHK-21 cells were infected with the viruses, viral titers from day 1 to day 6 post infection were determined using foci formation assay (FFA). We found that the recombinant DENV etDENV produced a comparable kinetic to that of wild type DENV 16681 (Additional file 1: Figure S1) up to 6 days. Once prepared, the viral titration was performed as follows. Supernatant of transfected infected cells was collected for FFA at 7 days post transfection. For FFA, the supernatant was incubated with BHK-21 cells in 96 well plates for 2 h, and subsequently 200 μl of 1.5 % carboxymethylcellulose (CMC) and 2 % FBS in DMEM was added to cover the cells, and the cells were incubated at 37 °C for 3 days. The infected BHK-21 cells were then washed with PBS 3 times and fixed with 3.7 % formaldehyde for 10 min at room temperature. The cells were permeabilized by 1 % Triton X-100 in PBS for 10 min and washed with PBS 5 times. The cells then were incubated with 1:1,000 in PBS anti-DENV E protein (EMD Milipore). Thereafter, the cells were washed with PBS 5 times before incubation with goat anti-mouse conjugated horseradish peroxidase (HRP) antibody at a 1:1,000 dilution for 45 min at 37 °C. The foci were visualized through SigmaFAST™ DAP (Sigma) and counted under microscopy to determine viral titter.

### Viral infection

In ever experiment, wild type DENV2 strain 16681 [35] or His-FLAG-tagged DENV was added into host cells at a multiplicity of infection (MOI) of 1, with an exception of the experiment with PMA treatment, in which virus with MOI of 5 was used. Cells were incubated at 37 °C for 90 min to allow incorporation of virus in to cells before being washed twice with PBS to remove excess virus. After that, DMEM medium supplemented with 2 % heat-inactivated FBS was added into the cells. Cells were further incubated at 37 °C for for time points indicated in the figures.

### Computational prediction of phosphorylation sites in Dengue NS5 protein

Bioinformatics tools used for screening of the possible PKC sites, Scansite™ 2 [18], NetPhosK 2.0 [19], and KinasePhos 2.0 [20], were used for predicting phosphorylation sites in Dengue NS5 protein. A high stringency level was set for Scansite™, whereas a 0.9 threshold (high) was used for NetphosK, and a 100 % specificity was used for KinasePhos.

### Sequence and structure analysis

To identify the conservation among flaviviruses, DENV1 (P27909), DENV2 (AHG23127), DENV3 (P27915), DENV4(P09816), West Nile virus (P06935), Japanese Encephalitis virus (P27395), yellow fever virus (AAC35930), St. Louis encephalitis virus (P09732), Omsk hemorrhagic fever virus (AAR98531), Zika virus (W8R1T1), Gadgets Gully virus (A0EKU2), Kama virus (W5VRZ2) and tick-borne encephalitis virus (Q01299) NS5 sequences were compared by BioEdit 7.2.5. The phospho-conservative site was shown in crystal structure of NS5 (PDB ID: 4V0R).

### Cell viability assay

To assess cytotoxicity of Bisindolylmaleimide I (BisI) Hydrochloride (Cell signaling) to HepG2 cells, the cells were seeded in 96-well plates at a density of 10^4^ cells/well in DMEM medium supplemented with 10 % heat-inactivated FBS for 24 h before BisI treatment. BisI at concentrations of 0.001 μM, 0.01 μM, 0.1 μM, and 1 μM in DMEM medium supplemented with 10 % heat-inactivated FBS were added onto the cells. DMSO at a final concentration of 0.5 % served as a vehicle control. At 24, 48, and 72 h post-treatment, cell viabilities were assessed by a MTT assay, following the manufacturer protocol. To assess cytotoxicity of siRNA to HepG2 cells, the cells were seeded in a 96-well plate at a density of 10^4^ cells/well in DMEM medium supplemented with 10 % heat-inactivated FBS for 24 h before being transfected with PKCα siRNA at 24, 48, and 72 h post-transfection, MTT was added as described above.

### Viral replication assay in BisI-treated cells

HepG2 cells (5.5 X 10^5^ cells) were seeded into culture dishes for 24 h before being treated with various concentrations of BisI, i.e. 0.001 μM, 0.01 μM, 0.1 μM, and 1 μM. BisI was added into the cells 30 min before viral infection. BisI was kept constant in the medium during infection with DENV2 at MOI of 1. This was followed by an incubation for 90 min to allow internalization of virus into cells. After the excess virus was removed, and the cells were washed twice by PBS, BisI in DMEM supplemented with 2 % FBS was added into cells and further incubated for the desired times, before the cells were harvested for detection of intracellular viral RNA by RT-qPCR.

### Viral replication assay in PKCα–knockdown cells

PKCα siRNA (Santa Cruz biotechnologies, SC-29449) or Silencer® Negative control siRNA (Thermo, AM4611) were transfected into HepG2 cells using Lipofectamine® RNAiMAX reagent (Invitrogen) following the manufacturer’s protocol. HepG2 cells (5.5 × 10^5^ cells) were seeded for 24 h before siRNA complexes were added. Twenty nM of siRNA were effective for suppressing PKCα expression. At 24 h post-transfection, DENV2 at MOI of 1 was added into the HepG2 cells and the culture was further incubated for 90 min to allow internalization of virus into the cells. Then, the excess virus was removed, and the cells were washed twice with PBS. Finally, DMEM supplement with 2 % FBS was added into the cells, and the cells were further incubated for another 24 h. Cells were collected for detection of intracellular viral RNA by RT-qPCR and viral proteins by immunoblots.

### Intracellular viral RNA quantification by RT-qPCR

Total RNA was extracted from DENV-infected cells by Trizol reagent (Invitrogen), according to manufacturer’s protocol. The reverse transcriptase reaction was set up using iScriptTM select cDNA synthesis kit (Bio-Rad) for first-strand cDNA synthesis. One microgram of total RNA was used as a template for all RT reaction. The reactions were performed with random primers. qPCR reactions were carried out using 2X KAPPA SYBR Green (KAPPA Biosystem). Amplification and detection were performed using an Applied Biosystems 7500 Real-time PCR system (ABI, California). qPCR conditions were 95 °C for 3 min, followed by 40 cycles of 95 °C for 3 s and 60 °C for 1 min.

Primers are:

NS5-Forward primer 5’-TCCATACATGCTAAACATGA-3’

NS5-Reverse primer 5’-GGGATTTCCTCCCATGATTCC-3’

β-actin-Forward prime 5’-TCTTCCAGCCTTCCTTCCT-3’

β-actin-Reverse primer 5’-AGCACTGTGTTGGCGTACAG-3’

### Western blot analysis

HepG2 cell lysates were suspended in RIPA buffer solution containing 50 mM Tris-base pH 7.4, 150 mM NaCl, 1 % triton X-100, 0.1 % SDS, 1 % sodium deoxycholate, 1 mM EDTA, 1 mM Na_3_VO_4_, 1 mM NaF, 1 mM β-glycerophosphate, 0.5 % NP40, 1 mM PMSF and 7× protease inhibitor cocktail. Protein samples were mixed with 6× protein loading dye, boiled and separated using SDS-PAGE. Gel was run for 2 h at 120 V before the proteins were transferred onto PVDF membrane by semi-dry blotting for 30 min at 10 V. After the transfer, nonspecific binding was blocked using 5 % skim milk for 1 h at room temperature. To determine inhibitory activity of PKC by BisI, after 24 post-infection, primary and secondary antibodies were rabbit antibodies against p-PEA15, PEA15, and goat anti rabbit antibody conjugated with horseradish-peroxidase (HRP) (Thermo scientific), respectively. To test whether PKCα siRNA can knock down PKCα after 24 and 48 h post-transfection, expressions of PKCα were detected by using mouse antibody specific to PKCα, and goat antibody specific to GAPDH (loading control) (both were from Santa cruz biotechnologies) as primary antibodies and goat anti-mouse (Novagen) and mouse anti-goat antibodies conjugated with HRP (Santa cruz biotechnologies) as secondary antibodies. To verify expression of NS5, rabbit antibody against NS5 (Thermo scientific) was used, followed by goat anti-rabbit antibody conjugated with HRP as secondary antibodies. The antigen-antibody complexes were detected by using chemiluminesence detection (ECL, Amersham Pharmacia Biotech).

### Determination of in vivo phosphorylation of NS5 by PKC

In this test, we performed infection of the recombinant virus on BHK-21 cells, due to the relatively high expression level of viral proteins obtained from this cell line. BHK-21 cells were treated with 1 μM BisI before, at the time of, and after DENV infection as described above. At 24 h post-infection, cells were washed with cold PBS before extraction of total protein using CLB buffer (50 mM Tris HCl PH 7.4, 250 mM NaCl, 5 mM EDTA, 1 mM Na_3_VO_4_, 1 mM NaF, 1 mM β-glycerophosphate, 0.5 % NP40, 1 mM PMSF and 7X protease inhibitor cocktail). Three mg of cell lysate were used for FLAG-NS5 immunoprecipitation (IP) with 20 μl of anti-FLAG M2 affinity gel (Sigma) in 1 ml reaction according to the manufacturer’s instruction. IP products were separated by SDS-PAGE and transferred onto a PVDF membrane. The membrane was probed with rabbit anti-NS5 (Thermo scientific), mouse anti-phospho-Serine or anti-phospho-Threonine (Sigma) followed by incubation with goat anti-rabbit or goat anti-mouse IgG conjugated with HRP. The antigen-antibody complexes were detected by using chemiluminesence detection (ECL, Amersham Pharmacia Biotech).

### Statistical analysis

All experiments were performed in triplicate and data are presented as the mean ± standard deviation (SD). Statistical comparisons were performed using *Student’s t* test. *p*-value ≤0.05 was considered to indicate a statistically significant difference.

## Additional file

Additional file 1: Viral kinetics of wild type DENV 16681, and the recombinant DENV etDENV. (PDF 63 kb)
