# Supplementary material for: Inhibition of protein kinase C promotes dengue virus replication
Source: Virol J. 2016 Mar 1;13:35. doi: 10.1186/s12985-016-0494-6 (PMC4774189; doi:10.1186/s12985-016-0494-6)
Supplement: Additional file 1: — Viral kinetics of wild type DENV 16681, and the recombinant DENV etDENV. (PDF 63 kb) [file 12985_2016_494_MOESM1_ESM.pdf]

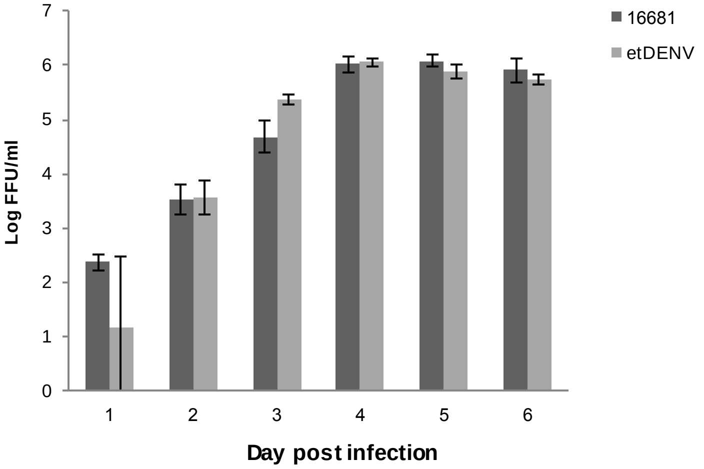

Supplementary Figure 1: Viral kinetics of recombinant DENV expressing His-FLAG tagged NS5 (etDENV) and the wild type DENV2 16681 (16681) on day 1 to day 6. The viral titers were determined by FFA using BHK-21 as host cells. The graphs show a representative experiment. Error bars represent S.D. from a triplicated experiments.
